# Diffusion tensor imaging for rehabilitation and prevention of lumbar degenerative diseases: a narrative review

**DOI:** 10.3389/fmed.2026.1959293

**Published:** 2026-10-22

**Authors:** Nan Zhang, Baohua Zhang, Zemin Zhao, Di Xu, Yang Xu

**Affiliations:** 1 Medical Imaging Department, 82nd Group Army Hospital, People’s Liberation Army, Baoding, Hebei, China; 2 North China University of Technology, Tangshan, Hebei, China

**Keywords:** chronic low back pain (CLBP), diffusion tensor imaging, lumbar degenerative disease (LDD), lumbar disc herniation (LDH), lumbar spinal stenosis (LSS), military personnel, paraspinal muscles, rehabilitation and prevention

## Abstract

Although objective imaging markers for rehabilitation assessment are available, lumbar disc herniation (LDH) and lumbar spinal stenosis (LSS) remain two common causes of chronic low back pain (CLBP). Diffusion tensor imaging (DTI) is a non-invasive imaging modality that allows estimation of water-molecule anisotropy and assessment of the microstructure of nerve roots and paraspinal muscles. Postoperative monitoring, preoperative prognostic assessment, evaluation of conservative treatment, detection of paraspinal muscle degeneration, and prevention of injuries during military training are examples of DTI applications in the rehabilitation and prevention of lumbar degenerative disease (LDD). There are two aspects of “prevention”: primary prevention, which concerns early warning of training injuries; and secondary prevention, which concerns the quality of rehabilitation to avoid long-term disability. Initial results suggest that DTI has the potential to identify progressive recovery of fractional anisotropy (FA) following surgery, act as a preoperative outcome predictor, evaluate the efficacy of conservative treatment, and detect age- and disease-related changes in the paraspinal muscle. These results have particular implications for military personnel, where DTI could be used to monitor training load and help reduce non-combat casualties. However, most findings remain preliminary and need to be confirmed in military populations.

## 1 Introduction

CLBP is one of the primary causes of disability and places a significant burden on the health care system (1). LDH (2) and LSS (3) are common causes of CLBP. This is a serious issue, especially among military personnel, with up to 81.7% of soldiers affected within one year (4). Back pain can have a major impact on a person's quality of life and significantly increase the risk of non-combat injuries, thereby reducing the effectiveness of combat preparation and action (5). Chronic pain is the primary cause of hospitalisation and disability among military personnel, resulting in more than 2 million medical visits, over 8 million days of limited-duty status, and up to $434 million in direct medical costs per year (6). Magnetic resonance imaging (MRI) is a widely used imaging modality for assessing lumbar spine pathologies; however, it cannot objectively quantify the severity of disc degeneration and has limited sensitivity for detecting early lesions (7). Moreover, conventional MRI is performed with the patient lying on their back and does not capture the dynamic biomechanical characteristics of the spine under load, which are important factors in triggering low back pain symptoms (8). Furthermore, conventional MRI cannot assess the functional integrity of neural tissue or provide microstructural information regarding axonal damage, demyelination, and the potential for regeneration (9). These limitations render conventional MRI poorly suited to monitoring the rehabilitation process and predicting functional outcomes.

DTI is an MRI technique that reflects the directionality of water-molecule diffusion (10). DTI quantifies the directional diffusion of water molecules in tissue and provides quantitative parameters; the most representative of these is FA, which reflects the microstructural integrity of tissue (11). Furthermore, FA values have been associated with the severity of clinical symptoms (12), functional impairment indices (13), and even postsurgical functional outcomes (14). Therefore, DTI may serve as a potential biomarker for functional evaluation and prognosis, as well as an anatomic imaging modality.

Past research into DTI and its use in LDD has mainly focused on the precise localisation and quantification of the affected nerve root (15) and on its use to assist surgical decision-making (16). We have already provided an overview of the use of DTI for diagnosing LDD (17). However, rehabilitation monitoring and prognosis are also key components of clinical decision-making and are equally central to patient management. Given the relatively concentrated and well-established research evidence on DTI in LDH and LSS, this narrative review will focus on common LDDs (LDH and LSS) to provide a comprehensive summary of the potential of DTI in rehabilitation monitoring and the development of preventative strategies. This review discusses two levels: primary prevention and secondary prevention. In particular, the focus is on the use of DTI in military and primary care settings, as these settings are particularly well-suited to special populations, including military personnel, who require objective, quantitative assessments of neurological recovery.

### 1.1 Search strategy and study selection

Given the narrative scope of this review, we did not perform a formal systematic review or meta-analysis; however, we adopted a clear and reproducible literature search strategy. A systematic search was conducted in PubMed and Web of Science from inception to August 2026. The search combined terms from three domains using Boolean operators (AND between domains and OR within each domain): Domain 1 (imaging): “diffusion tensor imaging” OR “DTI”; Domain 2 (disease): “lumbar disc herniation” OR “lumbar spinal stenosis” OR “lumbar degenerative disease” OR “lumbosacral radiculopathy”; Domain 3 (application): “rehabilitation” OR “recovery” OR “physical therapy” OR “conservative treatment” OR “postoperative” OR “paraspinal muscles” OR “military training” OR “prevention.” Searches were limited to English-language publications and human studies, with animal studies included only when they provided complementary histopathological evidence. One author screened titles and abstracts; all candidate full-text articles and records with unclear relevance during the initial screening were independently cross-checked by at least two co-authors, with disagreements resolved through discussion. We included original research and systematic reviews reporting DTI parameters (FA, MD, AD, and/or RD) in patients with LDH or LSS, with clinical outcomes or imaging–clinical correlations relevant to rehabilitation or prevention. Case reports, conference abstracts, editorials, and opinion pieces were excluded. Grey literature (e.g., doctoral dissertations) and unpublished trial registry records were considered only when identified through hand-searching or author knowledge; such sources are explicitly cited as such in the text or reference list. Reference lists of included articles were hand-searched for additional studies. Findings were organized into three thematic sections: nerve root assessment, paraspinal muscle evaluation, and military applications.

## 2 The principles of DTI and its key parameters in LDD rehabilitation assessment

### 2.1 Basic principles of DTI

The imaging principle of DTI involves the use of diffusion-sensitive gradients in different directions to quantitatively analyse the random diffusion of water molecules in different tissues and the degree of diffusion restriction (18). In neural tissue, water diffusion is greater along the long axis of the axons than across them, and diffusion along the long axis is relatively unrestricted, a property referred to as “anisotropy” (19). DTI technology uses diffusion-sensitive gradients in at least six spatial directions to acquire three-dimensional diffusion signals from the diffusion of water molecules and convert them into visualised quantitative indicators (20). Given its strong sensitivity to microstructure, DTI could be used to detect early or subtle pathological changes that conventional MRI cannot detect, providing an additional imaging approach for assessing degenerative lumbar spine diseases during rehabilitation.

### 2.2 Clinical interpretation of the DTI multiparameter model in the rehabilitation of lumbar nerve roots

The main quantitative DTI measures include FA, mean diffusivity (MD), axial diffusivity (AD) and radial diffusivity (RD). These parameters reflect the microstructural state of neural tissue from different perspectives. FA quantifies the anisotropy of water-molecule diffusion and ranges from 0 to 1. Higher FA values represent more directional diffusion and more regular fiber alignment, whereas a lower FA value close to 0 indicates diffusion that is more likely to be isotropic, suggesting disruption of fiber structure or disorganized fiber orientation (21). Thus, FA can sensitively detect nerve fiber integrity. During rehabilitation, an increase in FA may reflect recovery-related changes in nerve root microstructure and may provide a useful imaging parameter for monitoring neural recovery (22). In diffusion-weighted imaging (DWI), the apparent diffusion coefficient (ADC) is the parameter most frequently used to represent the overall degree of water-molecule diffusion in tissues. Trends in ADC changes are in good agreement with MD, which indirectly reflects the level of tissue edema and cellular microstructure. Clinically, these measures are frequently combined for a comprehensive evaluation of nerve injury and rehabilitation (23).

Compression of the lumbar nerve roots causes changes in DTI values. Hughes et al. (24) performed DTI examinations in elderly patients with L5 nerve root compression caused by spinal canal or intervertebral foramen stenosis and found that, compared with the uncompressed L4 or S1 nerve roots, FA was significantly reduced in the compressed nerve root, while MD, AD, and RD were significantly elevated in the proximal segment. These parameter changes provide imaging evidence for understanding the pathophysiological processes following nerve root compression, including axonal injury, demyelination, and edema.

It is important to note that, regardless of whether the compression results from spinal stenosis or a herniated disc, the trends in DTI parameters of the compressed nerve itself are similar. However, the specific patterns of parameter changes during rehabilitation remain to be fully established. In a prospective study of physical therapy in patients with LDH, Badr et al. (22) reported that FA increased and ADC decreased after treatment, accompanied by clinical symptom improvement. This study did not report MD, AD, or RD values. Therefore, the combined patterns of multiple DTI parameters in the context of rehabilitation should be interpreted with caution, and current evidence is insufficient to define distinct parameter combination patterns for guiding personalised rehabilitation decisions.

## 3 The application of DTI in postoperative rehabilitation for LDD

Surgical decompression is the fundamental approach to relieving nerve root compression and restoring nerve function; however, the postoperative microstructural recovery of the nerve root and its relationship with functional prognosis remain important areas of clinical attention (25). Recent studies have shown (26) that imaging techniques such as DTI can be used to dynamically track the recovery of postoperative nerve root microstructure, and that changes in microstructural parameters, such as FA, are significantly correlated with clinical outcomes including postoperative pain relief and functional improvement. Therefore, DTI offers a promising approach to postoperative rehabilitation assessment, with potential for dynamically monitoring nerve root recovery and providing preliminary prediction of functional outcomes, although further validation is needed**.**

### 3.1 Dynamic monitoring of postoperative nerve root microstructural recovery

Prospective longitudinal studies have shown progressive recovery of FA in compressed nerve roots after surgery. Li et al. (27) conducted a prospective longitudinal study of 19 patients with LDH who underwent percutaneous transforaminal endoscopic discectomy (PTED). FA values of compressed nerve roots were measured at 3, 30, and 90 days preoperatively and postoperatively. Before surgery and at 30 days after surgery, the FA value on the affected side was significantly lower than that on the healthy side (Group L5: 0.354 ± 0.031 before surgery vs. 0.448 ± 0.037, 0.408 ± 0.035 after surgery vs. 0.462 ± 0.036, *p* < 0.01; similar trend in Group S1). By 90 days after surgery, the FA value on the affected side had recovered to a level with no significant difference from the healthy side (Group L5: 0.421 ± 0.018 vs. 0.442 ± 0.034, *p* = 0.38; Group S1: 0.437 ± 0.024 vs. 0.445 ± 0.025, *p* = 0.134). This study confirmed for the first time that recovery of neural root FA is a dynamic and progressive process, and that by 90 days after surgery, the FA value had essentially reached the level of the healthy side. These findings suggest that FA evaluation before and early after surgery may provide useful reference information for surgical timing and prognostic assessment, although they are based on a small cohort and require further validation.

In addition to the overall recovery trend of FA, another study further revealed the regional recovery characteristics of postoperative nerve root microstructure from a segmentation-analysis perspective. Monti et al. (28) performed DTI follow-up in 7 patients with LSS before and after implantation of an interspinous process device (IPD), and found that although there was no significant change in overall FA in the treated segments, intraforaminal FA increased (*p* = 0.0229), while MD, AD, and RD decreased in the anterior segments of the intervertebral foramina (*p* < 0.05), and the visual analog scale (VAS) improved significantly (*p* < 0.0001). This finding suggests that DTI may capture regional repair of postoperative nerve roots, and that segment-specific FA changes may serve as an objective monitoring indicator for neural functional reconstruction.

Overall, the dynamic recovery of FA observed by Li et al. (27) is consistent with findings from the systematic review (29). Monti et al. (28) further extended these findings by demonstrating regional recovery patterns from a spatial perspective. Meanwhile, the systematic review by Pesesse et al. (30) demonstrated the value of DTI for such assessments. This review combined the results of 29 trials involving 1,056 patients and showed that DTI is a reliable and discriminative imaging method for the effective assessment of symptomatic lumbosacral nerve roots. Furthermore, DTI, particularly FA, was more consistent and reliable than DWI in determining the affected nerve root. These findings support the use of DTI parameters for dynamically monitoring postoperative neural recovery.

### 3.2 Preoperative DTI parameters as potential predictors of postoperative functional outcomes

In a prospective single-centre study of 117 patients with LDH, Wu et al. (14) reported that the preoperative FA value on the affected side was strongly associated with surgical success (AUC = 0.900, 95% CI: 0.829–0.971). Using the maximum Youden index, the suggested cutoff for affected-side FA was >0.212, which showed a sensitivity of 0.99 and specificity of 0.77 for predicting favourable outcomes. Despite these encouraging results, these DTI thresholds come from a single centre study, and will need external validation using independent patient cohorts and/or different surgical methods before they can be used in routine clinical decision making.

Among the 68 patients examined in the study of Lu et al. (31) to evaluate the diagnostic value of DTI in LDH, close correlation was found between the value of DTI parameters and the severity of clinical symptoms. DTI was sensitive to 80.43% and specific to 68.18% in the diagnosis of nerve root compression with a diagnostic concordance of 76.47% with respect to surgery. Correlation analysis showed that the FA value on the affected side was significantly negatively correlated with the VAS pain score (*r* = −0.614) and the Oswestry Disability Index (ODI) (*r* = −0.498, *p* < 0.05), while the ADC value was significantly positively correlated with both the VAS pain score (*r* = 0.623) and ODI (*r* = 0.535). From the perspective of the extent of nerve damage, this study provides a basis for using DTI parameters to evaluate the severity of a patient's condition before surgery and thus indirectly provides evidence for prognosis.

In terms of surgical planning, An et al. (32) conducted a retrospective case-control study of 128 patients with degenerative disease at the L4–L5 level to assess the usefulness of preoperative DTI in guiding extreme lateral interbody fusion (XLIF). In the conventional group (68 patients), the puncture site was located at the junction of zones II and III in each case. In the DTI group (62 patients), personalised entry points were determined based on the three-dimensional relationship between the psoas major muscle and nerve pathways using DTI three-dimensional reconstruction. The incidence of immediate postoperative complications in the DTI group was significantly lower than that in the conventional group (thigh pain/numbness: 2 cases vs. 10 cases, *p* = 0.031; lower limb motor dysfunction: 1 case vs. 8 cases, *p* = 0.033). The present study indicated that DTI-based preoperative planning might help minimise the risk of nerve injury, increase surgical safety, and indirectly contribute to better postoperative functional outcomes.

Taken together, the available evidence suggests that preoperative DTI assessment may assist in identifying patients at higher risk of poor outcomes (14, 31, 32), although the current data are largely derived from single-centre studies and require further validation. At present, these findings should be considered hypothesis-generating rather than practice-changing, and their clinical utility remains to be confirmed in prospective multicenter studies. Notably, the affected-side FA cutoff suggested by Wu et al. (14) (>0.212) and the single-root FA cutoff reported by Qian et al. (35) (<0.295) differ, and Qian et al.'s raw data show overlap between the good prognosis group (average FA 0.323) and the poor prognosis group (average FA 0.310). This inconsistency suggests that current DTI thresholds should not be used for clinical decision-making at present.

## 4 The application of DTI in non-surgical rehabilitation of LDD

The previous section has detailed the value of DTI in dynamically monitoring the recovery of nerve root microstructure and predicting prognosis during postoperative rehabilitation. However, in clinical practice, many patients with LDD receive conservative treatment, and there has long been a lack of objective imaging indicators for evaluating its effectiveness. This chapter will focus on the application of DTI in non-surgical rehabilitation, including changes in DTI parameters following physical therapy and its predictive value for treatment outcomes, with particular emphasis on its translational potential in the military population.

Common treatments for LDD include conservative approaches such as physical therapy, exercise therapy, and manual therapy, although their effectiveness is generally assessed using subjective rating scales. DTI provides a new tool for the objective assessment of the effects of conservative treatments on the microstructure of nerve roots.

### 4.1 Changes in DTI parameters following physical therapy

#### 4.1.1 The effects of multimodal physical therapy on DTI parameters and clinical symptoms

In the field of non-surgical rehabilitation, a 2024 prospective pilot study (22) recruited 14 patients with chronic unilateral lumbosacral radiculopathy (LSR) caused by paracentral L4-L5 or L5-S1 disc herniation. The patients underwent a 6-week course of multimodal physical therapy (MPT), including electrotherapy, manual therapy, and core stability training, three times weekly. The FA value of the compressed nerve root significantly increased from 0.226 to 0.268 (*p* < 0.001) after treatment, while the ADC value decreased significantly from 1.764 × 10^−3^ mm^2^/s to 1.616 × 10^−3^ mm^2^/s (*p* < 0.001). The VAS score decreased from 8.21 ± 0.87 to 2.46 ± 1.08, an improvement of 5.75 points that exceeded the minimum clinically important difference threshold of 2 points. Similarly, the Roland-Morris Disability Questionnaire (RMDQ) score decreased from 17.57 ± 3.27 to 4.64 ± 2.5, an improvement of 12.93 points that exceeded the minimum clinically important difference threshold by 3.5 points. To our knowledge, this study is the first to suggest that physical therapy can improve DTI parameters of compressed nerve roots in patients with LDH, and that FA values correlate with clinical symptom improvement, suggesting that FA may serve as an objective imaging surrogate marker for the efficacy of conservative treatment. These findings have significant clinical implications.

These studies suggest that DTI parameters are useful not only for evaluating outcomes in retrospective studies but also have potential clinical utility in prospective studies. A randomised controlled trial (NCT07558993, the MIRROR-LR study; registered trial, results not yet published) (33) is currently in progress and is the first to use DTI measurements of lumbar nerve root microstructural integrity (FA and other diffusion parameters) as a primary outcome measure to objectively compare the efficacy of Mirror Spinal Correction Training with that of standard conservative physical therapy. The trial is designed as a 12-week intervention involving adults with lumbar radiculopathy. Its aim is to evaluate dynamic changes in DTI parameters during the neurobiological recovery process and thereby address the limitations of traditional rehabilitation studies that rely on patient self-report. This research design represents a shift from an evaluation system that prioritises patients' subjective reports to a model incorporating both “subjective reports and objective imaging biomarkers”, helping to fully convey the clinical value of DTI in non-surgical rehabilitation assessment.

#### 4.1.2 The predictive value of DTI parameters for the outcomes of non-surgical treatment

The predictive value of DTI parameters for conservative treatment outcomes has been assessed in both retrospective and prospective settings. In a retrospective study of 364 LDH patients, Yang et al. (34) found that lower FA and higher ADC were independent risk factors for poor outcomes, and that the combination of DTI and conventional MRI parameters achieved better predictive performance (AUC = 0.877) than any single parameter (AUC = 0.767–0.803). This finding was further supported by a prospective study of LSS by Qian et al. (35), which used the lowest FA value from a single compressed nerve root and found that an FA threshold of <0.295 predicted poor prognosis with an AUC of 0.839 (sensitivity 77.3%, specificity 81.8%). Taken together, these studies suggest that DTI parameters, particularly FA, may help identify high-risk patients before treatment, although these findings are preliminary and require prospective validation before they can guide personalised rehabilitation planning.

#### 4.1.3 Potential clinical value of DTI parameters for military personnel

The above-cited studies of DTI application in non-surgical rehabilitation may have translational significance for the military population, but this application remains largely exploratory given the limited direct evidence currently available. Low back pain is much more common among military personnel than in the general population (36), due to high-intensity military training and specialised operating conditions. Furthermore, the aim of rehabilitation is not merely to alleviate symptoms but also to enable individuals to resume high-intensity training as quickly as possible. However, studies indicate that only approximately three-quarters (74.4%) of military personnel feel fully capable of performing their duties upon returning to duty after recovery, while the cumulative rate of recurrent injury within 1 year is as high as 37.9% (37). Currently, there is a lack of objective quantitative indicators in clinical practice to determine whether military personnel can “safely and effectively resume high-intensity training”. DTI parameters, particularly FA, show promise as exploratory imaging biomarkers for assessing rehabilitation progress and potentially guiding tiered rehabilitation training, although this application requires further validation in military-specific cohorts. For example, a study by Badr et al. (22) found that, in patients with LDH, the FA value of the compressed nerve root significantly increased after 6 weeks of multimodal physical therapy (MPT), while improvements in both VAS and RMDQ scores exceeded the minimum clinically important difference (MCID), suggesting that DTI parameters may be useful for monitoring the outcomes of conservative treatment. Future studies are needed to determine whether DTI-based rehabilitation criteria for military personnel can be established to enable more accurate and efficient individual rehabilitation management, thereby minimising the incidence of non-combat casualties and enhancing the combat preparedness of military units. However, at present, this remains a speculative application that requires substantial further investigation in military cohorts.

## 5 The application of DTI in the evaluation of paraspinal muscles in LDD

The previous sections have focused primarily on the use of DTI to assess the microstructure of nerve roots. However, the pathophysiological mechanisms underlying LDD involve not only nerve compression but are also closely associated with degeneration of the paraspinal muscles. Microstructural changes in the paraspinal muscles are equally important targets for rehabilitation monitoring and injury prevention. This chapter will explore the application of DTI in the assessment of paraspinal muscles.

### 5.1 Degeneration of the paraspinal muscles and LDD

The paraspinal muscles (namely the multifidus and erector spinae muscles) play an important role in the dynamic stability of the lumbar spine, in controlling trunk posture, and in carrying spinal loads (38). Meanwhile, LDDs, including LDH (39), spondylolisthesis (40), LSS (41), and scoliosis (42), are closely associated with degeneration of the paraspinal muscles. With advancing age and the progression of LDD, paraspinal muscles may exhibit degenerative changes such as muscle atrophy and increased fatty infiltration; these alterations are associated with low back pain and functional impairment (43). Although conventional MRI can evaluate muscle cross-sectional area and fat fraction, it cannot detect early microscopic structural changes in muscle fibres (44). Our team has previously summarised the basic principles and preliminary findings of DTI in the assessment of paraspinal muscles (17). Given the high prevalence of low back pain in the military population and their specific rehabilitation needs, this section will review current research findings and their potential translational applications in military training.

### 5.2 The principles and advantages of DTI in the assessment of paraspinal muscle microstructure

Skeletal muscle fibres are highly anisotropic: water molecules can diffuse relatively unobstructed along the long axis of muscle fibres, but diffusion in the perpendicular direction is restricted by myofibrils, the sarcolemma, and surrounding connective tissue. This anisotropy provides the physical basis for using DTI to evaluate the orientation and integrity of muscle fibres (45). Based on these structural features, DTI can be used to quantitatively analyse their microscopic morphology (46, 47). Recent investigations have shown that DTI can noninvasively characterise the microscopic structural properties of skeletal muscle and reliably identify microscopic changes in muscle tissue induced by intensive exercise (48, 49).

Unlike neural DTI, in muscle DTI, the FA value primarily reflects the orientation of muscle fibres and the integrity of fascicular structures; MD may be sensitive to changes associated with inflammation and tissue oedema, whereas AD and RD provide information about diffusion along and perpendicular to the predominant muscle-fibre orientation, respectively (50). Studies have shown (50) that DTI can distinguish between healthy and degenerated muscle and detect microstructural abnormalities even before the onset of fatty infiltration. In neuromuscular diseases, DTI parameters, such as FA and MD, have been shown to sensitively reflect microstructural changes in muscle, providing theoretical support for the application of DTI in assessing paraspinal muscle degeneration (45). Compared with conventional indices such as cross-sectional area and proton density fat fraction, DTI parameters can identify microstructural differences closely related to muscle function. Furthermore, monitoring dynamic changes in DTI parameters may allow the repair and rehabilitation processes following muscle injury to be assessed effectively. This benefit makes DTI as an objective imaging tool in the early evaluation of muscle injury and rehabilitation management for military training.

### 5.3 Research advances in the use of DTI for the evaluation of paraspinal muscles in LDD

Studies using different designs have provided evidence of DTI's usefulness in assessing paraspinal muscle degeneration. First, clinical studies have demonstrated that DTI can distinguish pathological from normal muscle. Li et al. (51) reported that individuals with persistent low back pain had reduced FA and increased ADC, and these changes were associated with pain severity and fatty infiltration. Second, DTI can also capture normal age-related physiological changes: Weedall et al. (52) showed higher FA and lower MD in older individuals, indicating that DTI can detect age-related structural changes beyond differences in fibre size alone. Third, mechanistic studies in animals support these clinical observations: Wu et al. (53) demonstrated a shift from type I to type II muscle fibres in a rat model, with FA changes related to functional deterioration; interestingly, the association between FA and the percentage of type I fibres (*r* = 0.493) is consistent with findings in humans (54). Collectively, these data suggest that DTI is sensitive to both disease-associated and age-related paraspinal muscle degeneration, although further long-term research is warranted.

### 5.4 Potential applications of DTI in the assessment of paraspinal muscles related to military training

Based on the above, DTI could be a feasible tool for assessing paraspinal muscle degeneration, although evidence specific to military populations remains limited. Building on this foundation, this section extends the application of DTI to the specialised field of military training medicine and explores its potential significance for objective monitoring of training loads, early injury warning, and rehabilitation decisions.

During military training, the paraspinal muscles are subjected to high-load conditions through repetitive lumbar flexion and extension movements and high-intensity weight-bearing exercises, and are therefore susceptible to training-related low back pain and muscle injuries (55). After weight-bearing training, the CSA and FCSA of soldiers' lumbar paraspinal muscles were significantly increased, with oedema observed in the muscle tissue (56). Thus, the microstructural state of the paraspinal muscles can be considered an important target for assessing military training loads and preventing training-related injuries. DTI is a functional imaging technique that offers the potential to provide a novel, quantitative, and non-invasive approach to objectively monitoring paraspinal muscle loading and predicting injury during military training.

Currently, there is very limited direct evidence regarding the use of DTI to assess paraspinal muscles in military personnel; however, the study by Berry et al. (57) provides relevant evidence. In this study, lumbar DTI scans were performed on active-duty Marines in four simulated combat positions: standing, weight-bearing, sitting, and prone with elbow support. The FA of the erector spinae muscles was found to be a significant predictor of the lumbar lordosis angle, lumbosacral extension angle, and sacral tilt angle (stepwise multiple linear regression, *p* < 0.05). FA values were inversely correlated with muscle fibre size, and soldiers with lower FA values exhibited poorer lumbar stability. This represents one of the few direct pieces of evidence on DTI assessment of paraspinal muscles in military populations to date, suggesting that DTI may have potential value in identifying individuals at high risk of training-related injuries, although these findings remain preliminary.

Based on the above evidence, the application of DTI in military training medicine could potentially develop in the following directions, although these applications remain speculative at this stage. By regularly monitoring changes in DTI parameters of paraspinal muscles before and after key training sessions, the effects of training on muscle microstructure could be objectively assessed, potentially allowing training load to be indirectly quantified and providing an objective basis for developing scientific and personalised training programmes. During rehabilitation, DTI parameters may provide objective information on muscle repair and potentially inform decisions about returning to high-intensity training; however, this application remains exploratory and requires further investigation in military cohorts. Future large-scale prospective cohort studies in military populations will be needed to validate the clinical utility of DTI parameters for preventing training-related injuries and to explore the establishment of DTI-based health assessment standards for the paraspinal muscles of military personnel.

In conclusion, the application of DTI in rehabilitation and prevention for LDD can be summarised into three progressive levels: (1) dynamic monitoring of nerve root microstructure and prediction of prognosis during postoperative and conservative treatment; (2) early identification and rehabilitation evaluation of paraspinal muscle degeneration; and (3) exploratory quantification of training loads and injury prevention in special populations, such as military personnel. These three levels span from nerve roots to paraspinal muscles, from disease treatment to functional rehabilitation, and from clinical settings to specialised environments, forming a comprehensive framework for the application of DTI in LDD rehabilitation medicine.

## 6 A critical analysis of the quality of the existing evidence

However, despite the above research, there are limitations in the current evidence, and the potential of DTI for the rehabilitation and prevention of LDD warrants critical evaluation. Several limitations should be considered, including small sample sizes, lack of control groups, short follow-up periods, possible publication bias, differences in DTI acquisition and analysis, and limited external validation of the proposed thresholds. These factors should be considered when interpreting the findings of this review. Beyond the study-specific limitations discussed below, a broader concern is the technical heterogeneity in DTI acquisition and analysis. DTI parameters such as FA and ADC can be influenced by scanner field strength (e.g., 1.5 T vs. 3.0 T), b-values, the number of diffusion-encoding directions, and echo time. Furthermore, post-processing methods vary considerably across studies, including ROI placement, the use of tractography vs. manual segmentation, and artefact correction. This technical heterogeneity highlights the importance of independently validating proposed thresholds before they are applied in clinical practice.

First, the cut-off value for preoperative FA used to predict surgical outcomes (FA > 0.212) was derived from a single-centre study. Whether this threshold is applicable to other surgical techniques or spinal segments remains unclear, and its generalisability would need to be confirmed by large-sample, multicentre prospective cohort studies (14). Second, findings from the small-sample trial (*n* = 14) without a control group suggest that physical therapy may improve DTI parameters, although this finding requires support from RCTs (22). Third, the segmental analysis of postoperative nerve root microstructural recovery (e.g., Monti et al., 28) was based on a very small sample (7 patients) with no control group; these findings need to be confirmed in larger studies. Fourth, the cross-sectional design of DTI studies examining paraspinal muscles in military cohorts does not allow causal inference, and the populations examined were limited to specific military branches, restricting generalisability (57). Moreover, most of these studies had short follow-up periods (1–3 months), and the dynamic changes in DTI parameters and long-term outcomes remain unclear. Therefore, the above evidence should be taken into account when using DTI parameters to inform clinical decisions.

Each study in Table 1 received an evidence-level grading based on study design, sample size, presence of a control group, and external validation of the findings. Studies with a prospective design, large sample size, and external validation were graded as High; studies with a prospective design but limited by a single-centre setting or small sample size were graded as Moderate; and studies with a retrospective, cross-sectional, or case-series design, or very small sample sizes, were graded as Low. Based on this scheme, the single-centre prospective prognostic study by Wu et al. (14) was graded as Moderate because its proposed FA thresholds have not yet been externally validated.

**Table 1:** Summary of key study evidence.

Author/Year	Study population	Research type	Key DTI findings	Clinical application value	Evidence level (study design and main limitations)
Li et al., 2019 (27)	Postoperative LDH (PTED)	Prospective longitudinal (*n* = 19)	Affected-side FA recovered to healthy-side level at 90 days postoperatively	Dynamic monitoring of postoperative nerve root recovery	Moderate (small sample size)
Wu et al., 2023 (14)	Preoperative LDH	Prospective prediction (*n* = 117)	Preoperative affected-side FA > 0.212 predicted favorable outcome (AUC = 0.90)	Preoperative prognosis assessment	Moderate (single-center, requires external validation)
Monti et al., 2025 (28)	Postoperative LSS (IPD)	Longitudinal case series (*n* = 7)	Significant FA increase in the intra-foraminal segment	Postoperative regional nerve repair assessment	Low (very small sample, no control group)
Badr et al., 2024 (22)	Conservative treatment for LDH	Prospective study(*n* = 14)	FA ↑, ADC ↓ after physical therapy;MD, AD, RD not reported	Objective evaluation of non-surgical rehabilitation efficacy	Low (no control group)
Yang et al., 2026 (34)	Conservative treatment for LDH	Retrospective analysis (*n* = 364)	Low FA and high ADC were independent risk factors for poor outcome	Prediction of conservative treatment outcome	Moderate
Qian et al., 2026 (35)	Conservative treatment for LSS	Prospective quantitative (*n* = 33)	Lowest FA < 0.295 predicted poor prognosis (AUC = 0.839)	Early identification of high-risk individuals	Moderate (single-center, external validation needed)
Li et al., 2024 (51)	CNLBP	Case-control (*n* = 73)	Paraspinal muscle FA ↓, ADC ↑	Early detection of paraspinal muscle degeneration	Moderate (cross-sectional design)
Berry, 2017 (57)	Active-duty military personnel	Cross-sectional study	Erector spinae FA correlated with lumbar spine stability	Training load monitoring and injury early warning in military training	Low (doctoral dissertation)
Hughes et al., 2019 (24)	Elderly patients with L5 nerve root compression	Cross-sectional study(*n* = 20)	FA↓ in compressed roots; MD, AD, RD↑ proximally (interpreted as axonal, myelin, and oedema changes)	Microstructural assessment of nerve root compression (preliminary)	Low (small sample, single-center)

Yang et al. (34) and Li et al. (51) are published in Chinese only; the interpretations presented here rely on the authors' translation. Wu et al. (14) may be difficult to access for some readers. These factors should be considered when interpreting the evidence levels and findings. Threshold variability: The FA thresholds reported in this table are derived from individual studies and vary considerably. Wu et al. (14) suggested a cutoff of >0.212 for favourable outcome, whereas Qian et al. (35) reported a cutoff of <0.295 for poor prognosis. However, in Qian et al.'s cohort, the average FA of the good prognosis group (0.323) was close to that of the poor prognosis group (0.310), and the single-root FA values also showed overlap. These inconsistencies highlight that current DTI thresholds are not validated for clinical decision-making.

A summary table (Table 1) captures the key evidence from the studies identified in this review.

Finally, although the findings presented above indicate that DTI could be a promising tool for the rehabilitation and prevention of LDD, its clinical use remains at the investigational stage. A number of the studies mentioned are still preliminary, and the suggested parameter thresholds have yet to be adequately tested across a variety of populations and contexts. For now, DTI parameters should be used with caution and not used alone to inform clinical decisions until standardised acquisition protocols and robust multicentre prospective studies are available. For now, their primary value lies in research settings and as an adjunct to conventional clinical assessment.

## 7 Summary and outlook

This review discusses DTI for rehabilitation and prevention of LDD. The Proposed research framework of DTI in the rehabilitation and prevention of LDD (Figure 1) remains at an early exploratory stage. Current studies suggest that DTI shows promise in objectively tracking nerve root recovery during rehabilitation, and preoperative parameters have shown preliminary associations with functional outcomes. However, these findings remain largely investigational; the proposed thresholds require external validation, and at present DTI may be viewed as a research tool with potential clinical applications rather than a validated decision-making aid.

**Figure 1:**
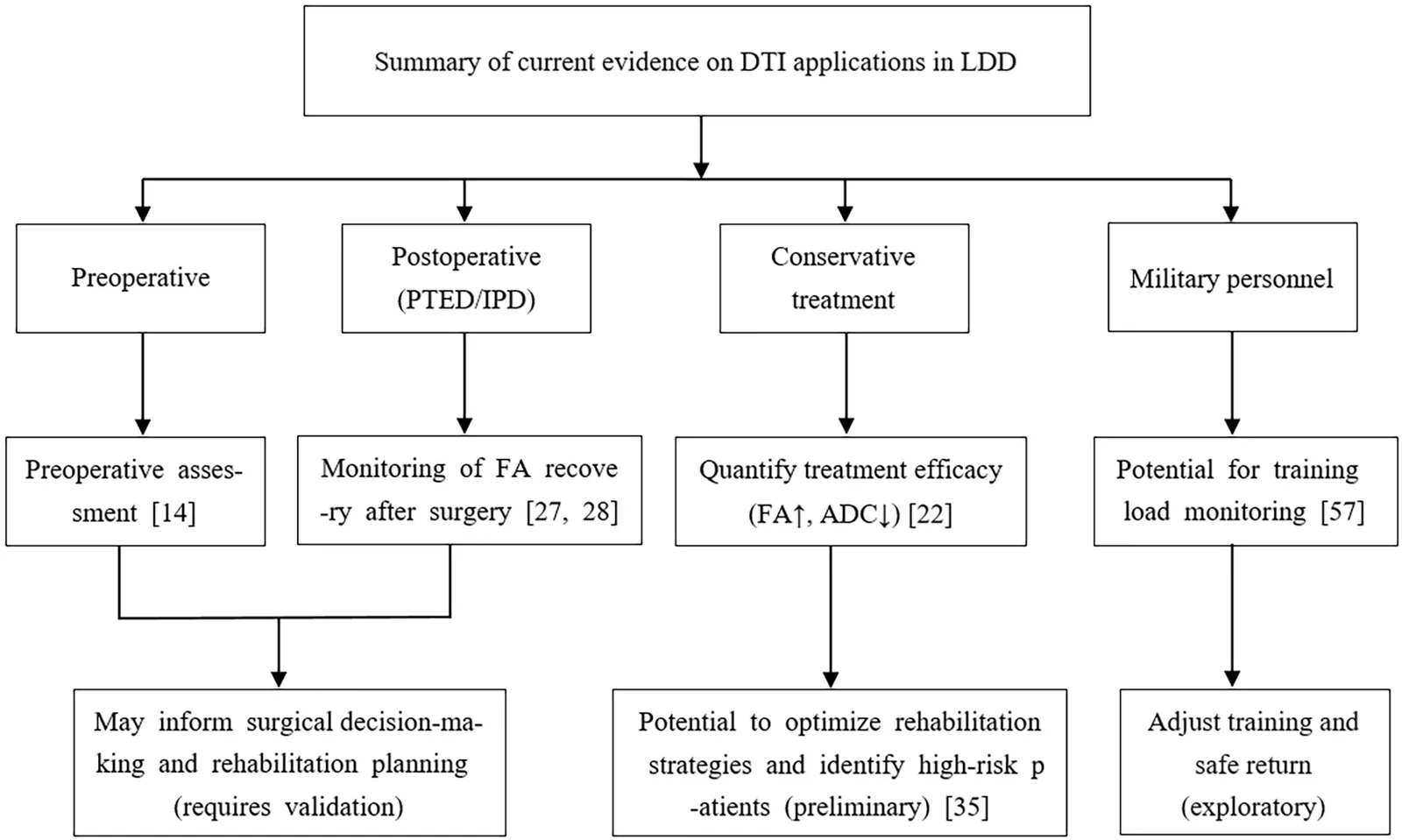
Summary of current evidence on DTI applications in LDD. This figure is not a validated clinical algorithm. It outlines four potential application areas: preoperative assessment, postoperative monitoring, conservative treatment evaluation, and military personnel (based on the referenced literature). Specific DTI thresholds are not shown because current evidence is insufficient to recommend any cut-off values. All applications require further validation. The flowchart domains correspond to the results presented in Table 1. Some studies included in this summary are published in Chinese only (Yang et al., 34; Li et al., 51) or may be difficult to access (Wu et al., 14). The FA thresholds reported in the literature show overlap (e.g., Wu et al., 14 vs. Qian et al., 35), and therefore no specific thresholds are recommended in this figure.

Future studies must prioritize large, multicenter, prospective cohort studies, with emphasis on filling the data gaps within the military. Such studies would be needed to determine whether DTI can make a major step from research tools to an approach for clinical rehabilitation assessments and become an objective decision making tool in military medicine for optimizing training programmes and minimising non-combat casualties. At present, however, this remains an area of active investigation rather than established practice.
